# 

**Published:** 1888-10

**Authors:** 


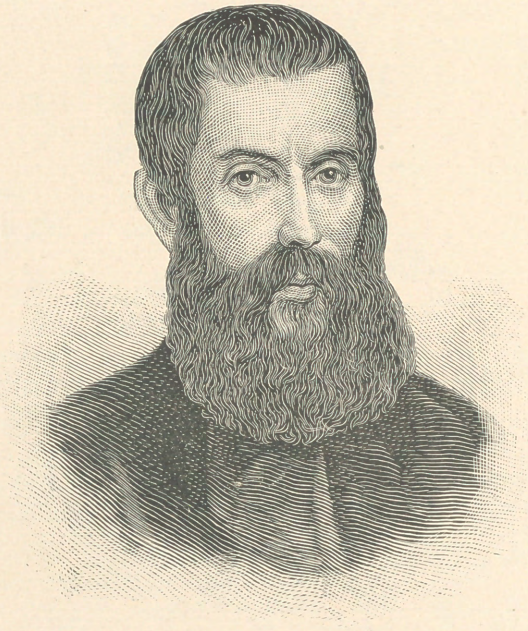

ANDREAS VESALIUS.

"Hippocrates has been styled the Father of Physic; Haller, the Father of Physiology; and Vesalius may fairly be considered as the Father of Human Anatomy."

### ANNOUNCEMENTS.

A Sanitary Convention will be held at Hastings, Mich., under the auspices of the State Board of Health, on Monday and Tuesday, December 3 and 4, 1888.

*Intercolonial Medical Congress of Australia.*—Preparations are already well advanced for the second congress, which will begin January 7, 1889, in Melbourne. Thomas N. Fitzgerald, F. R. C. S. I,. is the president-elect. The other officers have been nominated for the congress and for the sections, which number nine, with two sub-sections.

The announcement is made that members coming from Europa, America, or India will receive free passes over all Victorian railways, and all members of the congress will be entitled to return tickets at single fares, available for two months, over all the railways in Australia. Various shipping companies have consented to carry members of the congress at special rates for the sea passage.

Volume LVII.] OCTOBER, 1888. [Number 4.

INDEX MEDICUS

### The CHICAGO MEDICAL JOURNAL AND EXAMINER

EDITOR:
S. J. JONES, M.D., LL.D.

COLLABORATORS:

CHICAGO:

Professor E. ANDREWS, M.D., LL.D.
Professor J. BARTLETT, M.D.
Professor W. T. BELFIELD, M.D.
Professor F. BILLINGS, M.D.
Professor W. H. BYFORD, A.M., M.D.
Professor L. CURTIS, A.M., M.D.
Professor N. S. DAVIS, Jr., A.M., M.D.
Professor O. C. DE WOLF, A.M., M.D.
Professor C. FENGER, M.D.
Professor J. N. HYDE, A.M., M.D.
Professor E. F. INGALS, A.M., M.D.
Professor F. S. JOHNSON, A.M., M.D.
Professor H. A. JOHNSON, M.D., LL.D.
Professor C. W. PURDY, M.D.
Professor C. G. SMITH, A.M., M.D.
Professor E. WING, A.M., M.D.
Professor E. C. DUDLEY, A.B., M.D.

MILWAUKEE, WIS.:
Professor N. SENN, M.D., Ph.D.

WASHINGTON, D. C.:
S. O. RICHEY, M. D.

UNITED STATES ARMY:
Surgeon D. L. HUNTINGTON, M.D.

UNITED STATES NAVY:
Surgeon-General J. M. BROWNE, M.D. Medical-Director A. L. GIHON, A.M., M.D.

MARINE HOSPITAL SERVICE:
Surgeon S. T. ARMSTRONG, M.D., Ph.D.

